# Supplementary material for: Structures of p53/BCL-2 complex suggest a mechanism for p53 to antagonize BCL-2 activity
Source: Nat Commun. 2023 Jul 18;14:4300. doi: 10.1038/s41467-023-40087-2 (PMC10353994; doi:10.1038/s41467-023-40087-2)
Supplement: Supplementary file 1 — Supplementary Information [file 41467_2023_40087_MOESM1_ESM.pdf]

Supplementary Information for

Structures of p53/BCL-2 complex suggest a mechanism for p53 to antagonize BCL-2 activity

Hudie Wei[1,†], Haolan Wang[1,†], Genxin Wang[2,3], Lingzhi Qu[1], Longying Jiang[1,4], Shuyan Dai[1], Xiaojuan Chen[1], Ye Zhang,[1] Zhuchu Chen[1], Youjun Li[2,3], Ming Guo[1,*], Yongheng Chen[1,*]

[1]Department of Oncology, NHC Key Laboratory of Cancer Proteomics & State Local Joint Engineering Laboratroy for Anticancer Drugs, National Clinical Research Center for Geriatric Disorders, Xiangya Hospital, Central South University, Changsha, Hunan, 410008, China

[2]Hubei Key Laboratory of Cell Homeostasis, College of Life Sciences, TaiKang Center for Life and Medical Sciences, Wuhan University, Wuhan 430072, China

[3]Frontier Science Center for Immunology and Metabolism, Medical Research Institute, Wuhan University, Wuhan 430071, China

[4]Department of Pathology, Xiangya Hospital, Central South University, Changsha, Hunan, China

[†]These authors contribute equally to this work.

[*]To whom correspondence should be addressed. Tel: +86 731 84327542; Fax: +86 731 84327542; Email: yonghenc@163.com (Chen Y, lead contact), guomingxyyy@163.com (Guo M)

Supplementary Table 1. Data collection and refinement statistics

	Complex 1 (16-residue linker)	Complex 2 (19-residue linker)	Complex 2 (22-residue linker)
**Data collection**			
Wavelength (Å)	0.97918	0.97918	0.97918
Space group	P 21 21 21	P 21 21 21	P 21 21 21
Cell dimensions			
a, b, c (Å)	46.87, 70.48, 126.98	45.61 70.33 127.15	44.70 70.52 127.72
α, β, γ (°)	90, 90, 90	90, 90, 90	90, 90, 90
Resolution (Å)	61.62 – 2.62 (2.71-2.62)	25.51 - 2.52 (2.61 -2.52)	29.09 - 2.35 (2.44 - 2.35)
R_{merge}	0.25 (1.18)	0.088 (0.91)	0.17 (0.68)
I / σI	39.74 (2.83)	16.65 (2.63)	12.53 (2.82)
Redundancy	12.9 (13.0)	9.1 (9.6)	10.7 (7.4)
Completeness (%)	99.74 (99.85)	96.21 (99.64)	96.67 (82.17)
Wilson B-factor (Å^2)	55.25	57.88	39.36
**Refinement**			
Resolution (Å)	61.62 - 2.62	25.51 - 2.52	29.09 - 2.35
No. reflections	13194	13840	16801
R_{work} / R_{free}	0.22/0.28	0.23/0.28	0.21/0.25
No. atoms			
Protein	2528	2510	2611
Ligand/ion	1	1	1
Water	6	4	39
B-factors			
Protein	73.80	88.03	54.01
Ligand/ion	47.97	66.58	41.70
Water	53.88	63.36	46.59
R.m.s. deviations			
Bond lengths (Å)	0.003	0.008	0.005
Bond angles (°)	0.57	1.08	0.62
Number of TLS groups	14	16	15
Ramachandran favored (%)	97.55	96.33	96.72
Ramachandran outliers (%)	0.00	0.00	0.00
Rotamer outlier (%)	0.77	0.39	0.36
Clashscore	4.93	4.36	4.55
PDB code	8HLL	8HLM	8HLN

Supplementary Table 2. Sequences of pro-apoptotic BH3 peptides

BH3	Sequence
Bax	QDASTKKLSECLKRIGDELDS
Bak	PSSTMGQVGRQLAIIGDDINRRYDSE
Bid	DIIRNIARHLAQVGDSMDRSI
Bim	RPEIWIAQELRRIGDEFNAYYA
Puma	QWAREIGAQLRRMADDLNAQYERR

Supplementary Table 3. Primer Sequences

Primer name	Sequences (5'-3')
**Bak clone to pET28a:**	
**28a-BAKnhis-fw2**	GTTCTGTTTCAAGGCCCGGCTTCTGAGGAGCAGGTAG
**28a-BAKnhis-rev2**	GTGGTGGTGCTCGAGTTA ACCATTGCCCAAGTTCAGG
**Bax clone to pGEX-6p1:**	
**Bax-fw2**	TGTTCCAGGGGCCCCTGATGGACGGGTCCGGGGAG
**C21Bax-rev2**	ACGATGCGGCCGCTCGAGTCACTGCCACGTGGGCGTC
**vec6P1-FW**	GGGCCCCTGGAACAGAACTTC
**vec6P1-rev**	GGTCGACTCGAGCGGCCGC
**BCL-2 clone to pET-28a：**	
**28Chis-FW**	GGTATATCTCCTTCTTAAAGTTAAAC
**28Chis-REV**	CTCGAGCACCACCACCACCACCAC
**WtBCL2-FW1**	CTTTAAGAAGGAGATATACCATGGCGCACGCTGGGAGAAC
**WtBCL2-REV1**	GGTGGTGGTGGTGCTCGAGTCGCATGCTGGGGCCGTAC
**LC-BCL2-FW2**	CTTTAAGAAGGAGATATACCATGGCCCATGCCGGCCGCAC
**LC-BCL2-REV2**	GGTGGTGGTGGTGCTCGAGGCGCATGCTCGGACCATAC
**BCL2#3-FW3**	CTTTAAGAAGGAGATATACCATGTCACAAGATAATAGGG
**BCL2#3-REV3**	GGTGGTGGTGGTGCTCGAGAGCCGCGTTATTACCATATAAC
**BCL-2 clone to pGEX-4T1：**	
**GST-BCL2-fw**	GGTTCCGCGTGGATCCATGGCGCACGCTGGGAGAAC
**GST-BCL2-rev**	CGATGCGGCCGCTCGAGCATGCTGGGGCCGTAC
**BCL-2 clone to pQCXIH:**	
**pQC-HA-FW**	CAGGAATTGATCCGCGGCCGCATGTACCCATACGACGTCCCAG
**pQC-Bcl2-rev**	GAGGGGCGGAATTCCGGATCCTCACTTGTGGCCCAGATAG
**pQC-Bcl2-FW**	CAGGAATTGATCCGCGGCCGCATGGCGCACGCTGGGAGAAC
**pQC-Bcl2-rev**	GAGGGGCGGAATTCCGGATCCTCACTTGTGGCCCAGATAG
**BCL-2 clone to pCDH:**	
**PCDH-BCL2-FW1**	CATACGACGTCCCAGACTACGCTGAATTCATGGCGCACGCTGGGAG
**PCDH-BCL2-FW2**	GAAGATTCTAGAGCTAGCATGTACCCATACGACGTCCCAGACTAC
**PCDH-BCL2-REV**	CCTTCGCGGCCGCGGATCCTCACTTGTGGCCCAGATAG
**VEC-PCDH-HA-REV2**	GCTAGCTCTAGAATCTTCTATGGAG
**BCL-2 point mutation:**	
**bcl2-F104A-FW2**	GCCAGGCCGGCGACGACGCCTCCCGCCGCTACCGCC
**bcl2-F104A-REV2**	GGCGGTAGCGGCGGGAGGCGTCGTCGCCGGCCTGGC
**bcl2-R107A-FW8**	GGCGACGACTTCTCCCGCGCCTACCGCCGCGACTTCGC
**bcl2-R107A-REV8**	GCGAAGTCGCGGCGGTAGGCGCGGGAGAAGTCGTCGCC
**bcl2-Y108A-FW4**	ACGACTTCTCCCGCCGCGCCCGCCGCGACTTCGCCG
**bcl2-Y108A-REV4**	CGGCGAAGTCGCGGCGGGCGCGGCGGGAGAAGTCG
**bcl2-R146A-FW6**	GACGGGGTGAACTGGGGGGCGATTGTGGCCTTCTTTG
**bcl2-R146A-REV6**	CAAAGAAGGCCACAATCGCCCCCCAGTTCACCCCGTC

**p53-DBD clone to pGEX-6P1:**	
**GST-DBD-fw**	TGTTCCAGGGGCCCCTGATGCCCCTGTCATCTTCTGTC
**GST-DBD-rev**	ACGATGCGGCCGCTCGAGTCATTTCTTGCGGAGATTCTCTTC
**p53 clone to pQCXIH**	
**pQCXIH-53FL-fw**	TGCAGGAATTGATCCGCGGCCGCATGGAGGAGCCGCAGTCAG
**pQCXIH-53FL-rev**	AGAGGGGCGGAATTCCGGATCCTCAGTCTGAGTCAGGCCCTTC
**p53 point mutation**	
**53-S269E-FW7**	GTAATCTACTGGGACGGAACGAATTTGAGGTGCGTGTTTGTG
**53-S269E-REV7**	CACAAACACGCACCTCAAATTCGTTCCGTCCCAGTAGATTAC
**53-T284E-FW8**	CCTGGGAGAGACCGGCGCGAAGAGGAAGAGAATCTCCGC
**53-T284E-REV8**	GCGGAGATTCTCTTCCTCTTCGCGCCGGTCTCTCCCAGG
**53-D186R-FW**	CGCTGCTCAGATAGCCGTGGTCTGGCCCCTCCTC
**53-D186R-REV**	GAGGAGGGGCCAGACCACGGCTATCTGAGCAGCG
**53-L201E-FW**	CCGAGTGGAAGGAAATGAGCGTGTGGAGTATTTG
**53-L201E-REV**	CAAATACTCCACACGCTCATTTCCTTCCACTCGG
**53-L201R-FW3**	CCGAGTGGAAGGAAATCGTCGTGTGGAGTATTTG
**53-L201R-REV3**	CAAATACTCCACACGACGATTTCCTTCCACTCGG
**53-L188R-FW5**	CTCAGATAGCGATGGTCGTGCCCCTCCTCAGCATC
**53-L188R-REV5**	GATGCTGAGGAGGGGCACGACCATCGCTATCTGAG
**L188R-F9**	CGTGCCCCTCCTCAGCATCTTATCCGAG
**L188R-R9**	ACCATCGCTATCTGAGCAGCGCTCATG
**L210R-F10**	CGTCGTGTGGAGTATTTGGATGACAGAAAC
**L201R-RR**	ATTTCCTTCCACTCGGATAAGATGCTGAG
**Fused BCL-2-p53 clone to pET-28a:**	
**mutBCL2-53-F**	GAAGTTCTGTTTCAAGGCCCGTCACAAGATAATAGGGAAATAG
**mutBCL2-53-R**	GAACCAGAGAACCACCTCCACCGTTATTACCATATAACTCCAC
**19linker-1-fw1**	GTTCTGGAGGTGGTGGTTCTGGTGGATCTTCTGTCCCTTCCCAGAAAAC
**19linker-1-rev1**	GTTTTCTGGGAAGGGACAGAAGATCCACCAGAACCACCACCTCCAGAAC
**22linker-1-fw2**	GGAGTTATATGGTAATAACGGTGGATCTGGTGGAGGTGGTTCTCTGG
**21linker-1-rev2**	CCAGAGAACCACCTCCACCAGATCCACCGTTATTACCATATAACTCC
**p53 clone to pCDH:**	
**VEC-PCDH-FW**	GGATCCGCGGCCGCGAAGGATCTG
**VEC-PCDH-REV**	CATAAGCTTCTTGTCATCGTCGTCC
**PCDH-p53-FW**	CGATGACAAGAAGCTTATGGAGGAGCCGCAGTCAG
**PCDH-p53-REV**	CCTTCGCGGCCGCGGATCCTCAGTCTGAGTCAGGCCCTTC
**pCDH mutation:**	
**delGFP-FW**	ATGACCGAGTACAAGCCCACGGTGCG
**delGFP-rev**	GGTGGCGTCTAGCGTAGGCGCCGGTC

Supplementary Table 4. Sequences of siRNA

Bax siRNA	sense	GCCUCAGGAUGCGUCCACCAA
	antisense	UUGGUGGACGCAUCCUGAGGC
Bak siRNA	sense	GCUUCGUGGUCGACUUCAU
	antisense	AUGAAGUCGACCACGAAGC

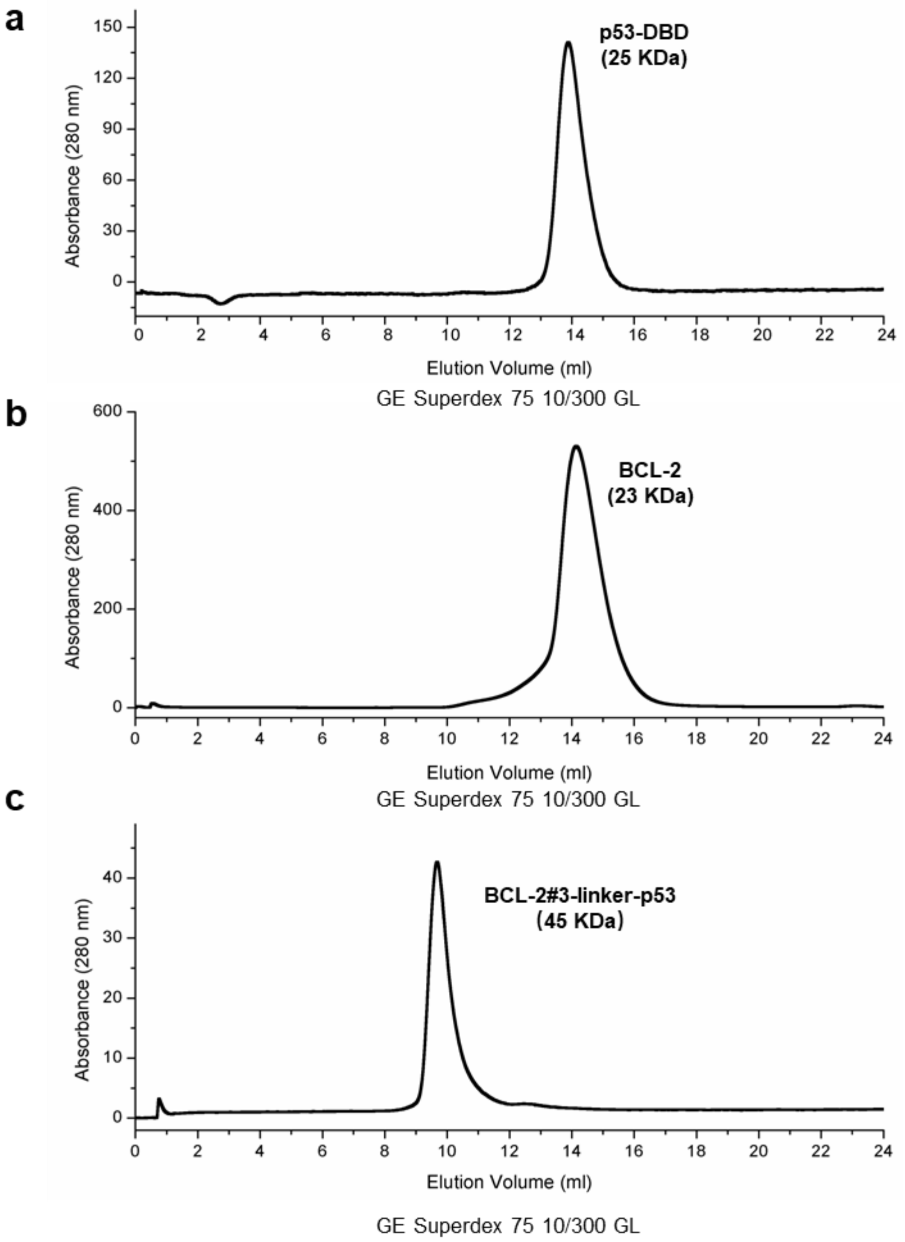

Supplementary Figure 1. Representative Gel filtration chromatography profiles of BCL-2 and p53-DBD proteins. **a**. p53-DBD; **b**. BCL-2; **c**. 22 residue-linked BCL-2#3-p53-DBD fusion proteins.

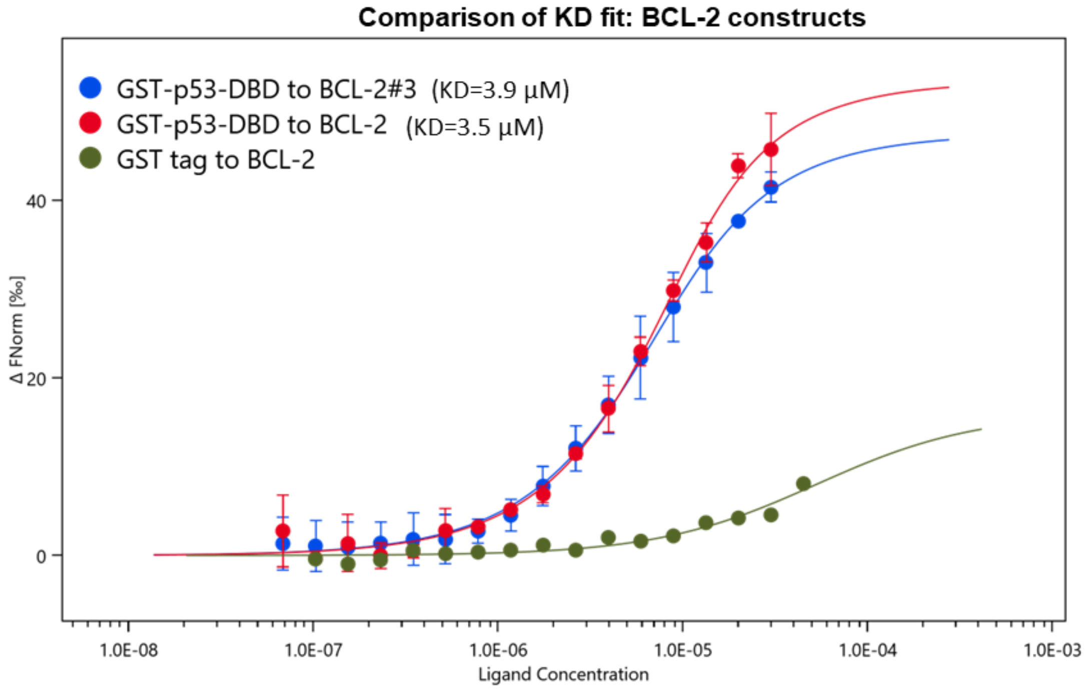

Supplementary Figure 2. The binding affinity of wild-type p53-DBD to BCL-2 or BCL-2#3 was analyzed by MST. A gradient dilution of GST-tagged p53-DBD (30 μM to 60 nM) was titrated against 50 nM RED-Tris-NTA dye-labelled 6×HIS-tagged BCL-2 at room temperature. The GST-tag control was tested. Curves were fitted and KD values of interactions were calculated using experimental data with a KD model by MO. Affinity Assay software. Data are represented as the mean ± SD of n=3 independent experiments. Source data are provided as a Source Data file.

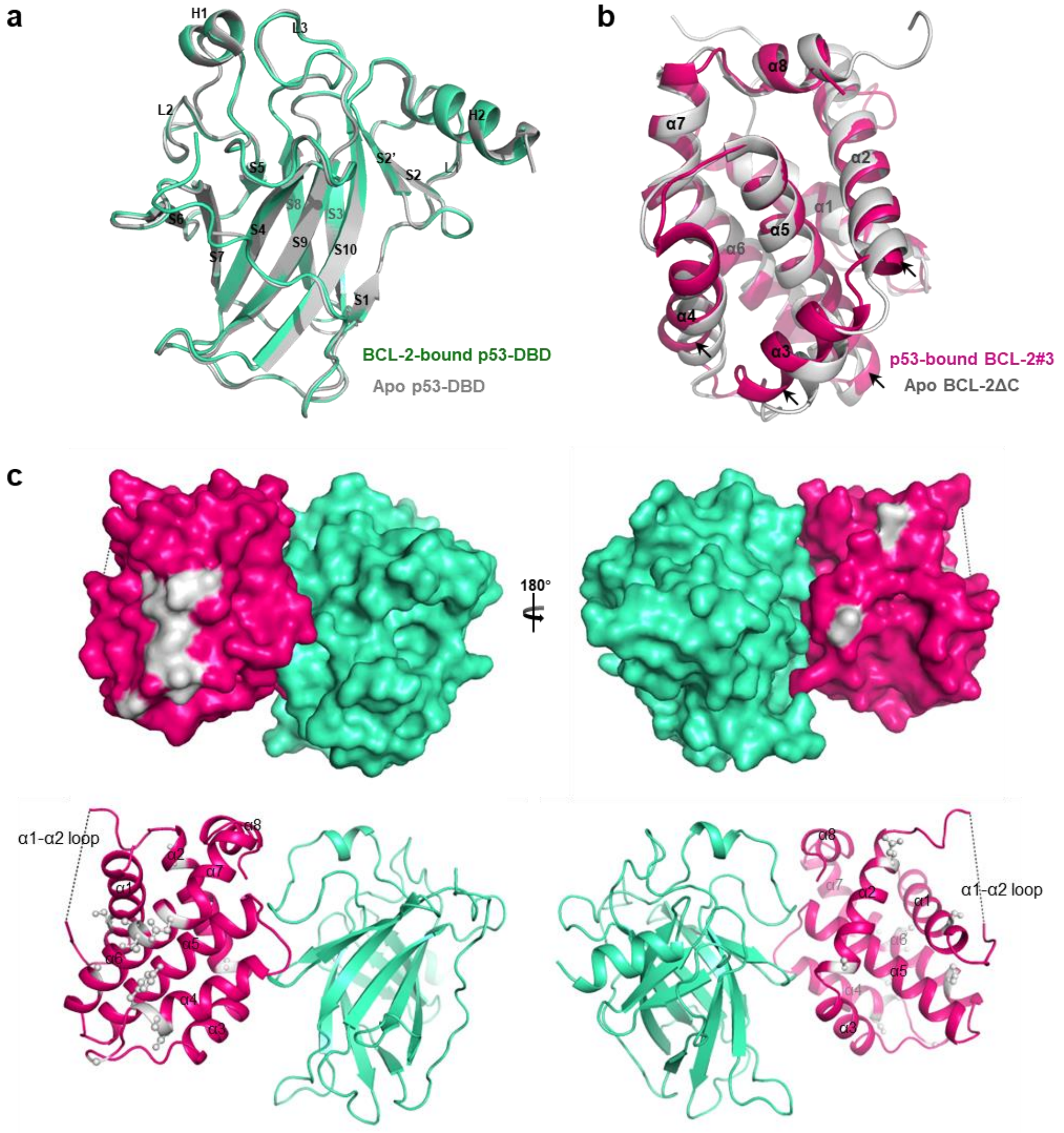

Supplementary Figure 3. Characterization of the crystal structure of the p53-DBD/BCL-2#3 complex. a. Alignment of the p53-DBD structure (colored green) in the p53-DBD/BCL-2#3 complex with the apo p53-DBD structure (PDB: 2OCJ, colored gray). The RMSD is 0.4 Å. The black arrows indicate the conformation changes. **b**. Alignment of the BCL-2#3 structure (colored hot pink) in the p53-DBD/BCL-2# complex with the apo NMR solution structure of BCL-2 (PDB: 1GJH, colored gray). The RMSD is 1.4 Å. **c**. Location of the modified residues in BCL-2#3. p53-DBD is colored green, and BCL-2#3 is colored hot pink. The modified surface residues of BCL-2#3 are colored gray. Some residues (amino acids 51-VEENRTEAP-59) of the α1-α2 loop that are not observed due to poor density are shown by black dotted lines.

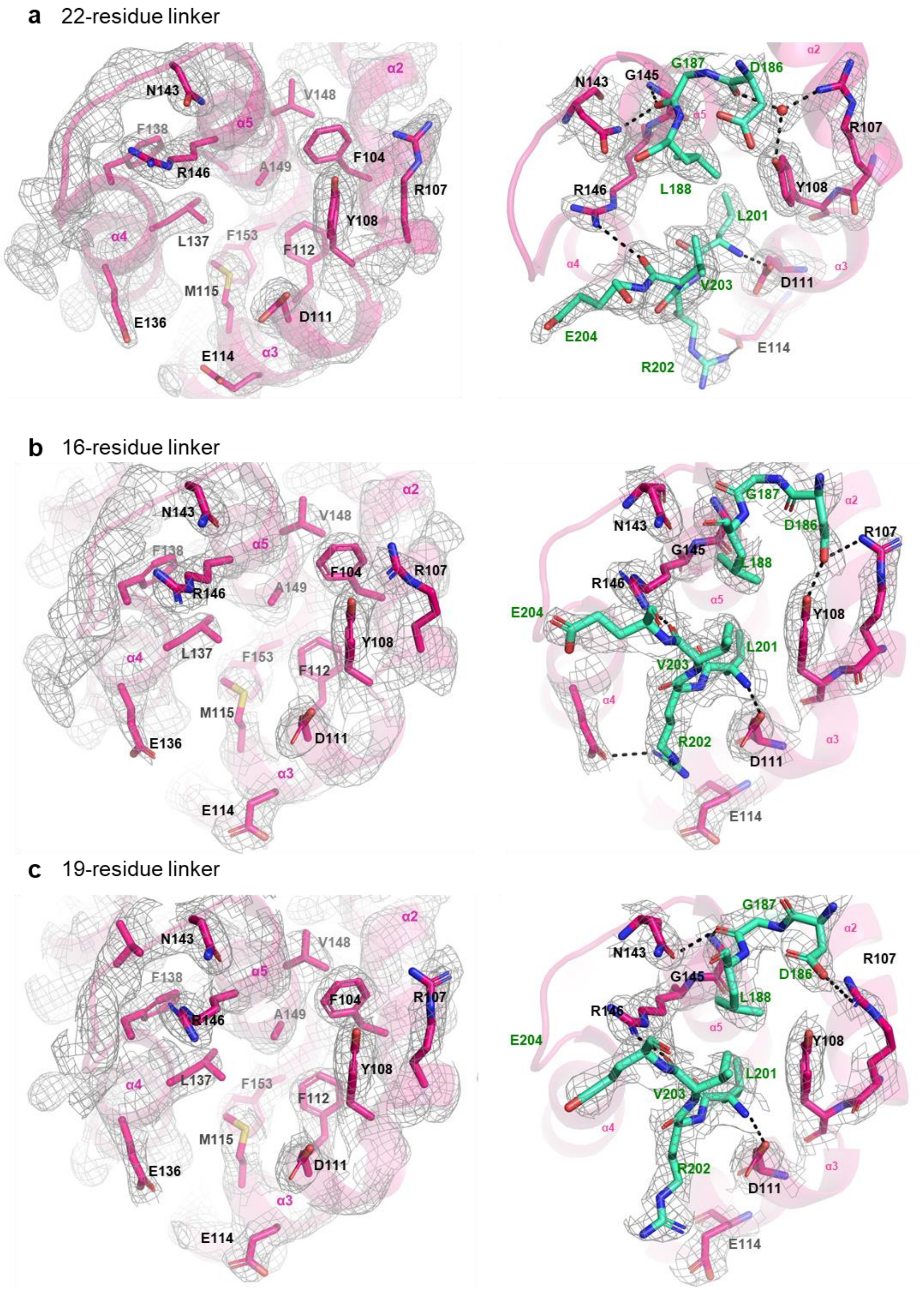

Supplementary Figure 4. The electron density map (2mFo-DFc) at 1σ shows the interfacial residues in the structures of the 22-residue (**a**), 16-residue (**b**) and 19-residue (**c**) linked fusion proteins. BCL-2 is colored hot pink and p53 is colored green. The interfacial residues are shown as sticks and labelled. Water molecules are indicated by red spheres.

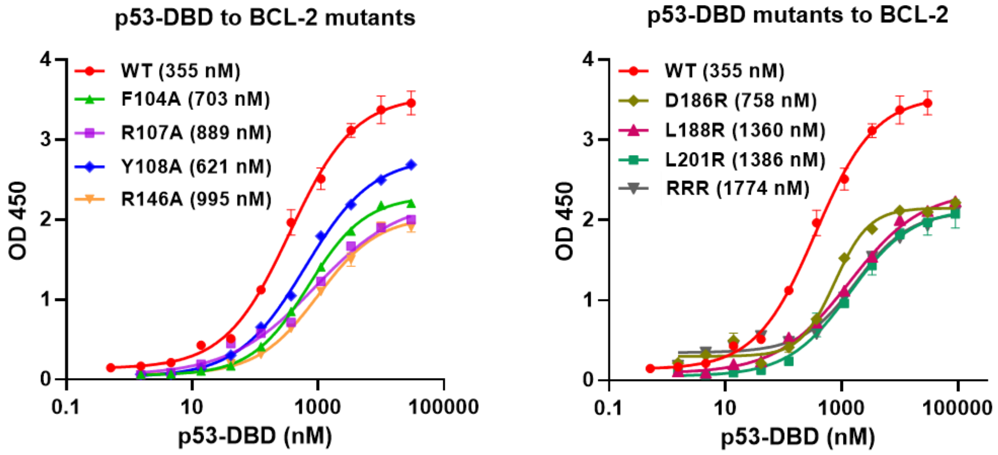

Supplementary Figure 5. ELISA assays detected the binding ability of GST-tagged p53-DBD to His-tagged BCL-2. Data are represented as the mean ± SEM of n= 4 independent experiments. The EC50 was generated by fitting the experimental data using a sigmoidal dose-response nonlinear regression model and is shown as indicated. Source data are provided as a Source Data file.

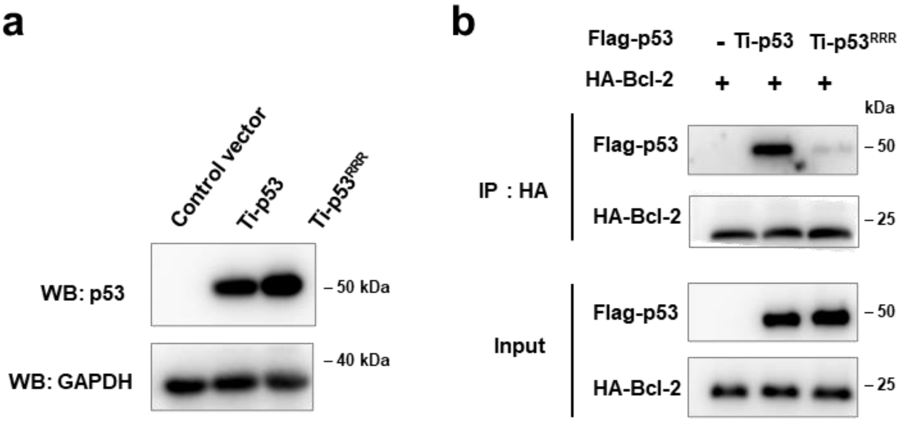

Supplementary Figure 6. Co-immunoprecipitation determining the interaction of full-length BCL-2 with Ti-p53 or Ti-p53^{RRR}. **a**. Western blotting detecting the protein expression of Ti-p53 or Ti-p53^{RRR} in p53-/- HCT116 cells. **b.** p53$^{-/-}$ HCT116 cells stably expressing Ti-p53 or Ti-p53^{RRR} were lysed and immunoprecipitated using anti-HA beads and subjected to western blotting as indicated. Source data are provided as a Source Data file.

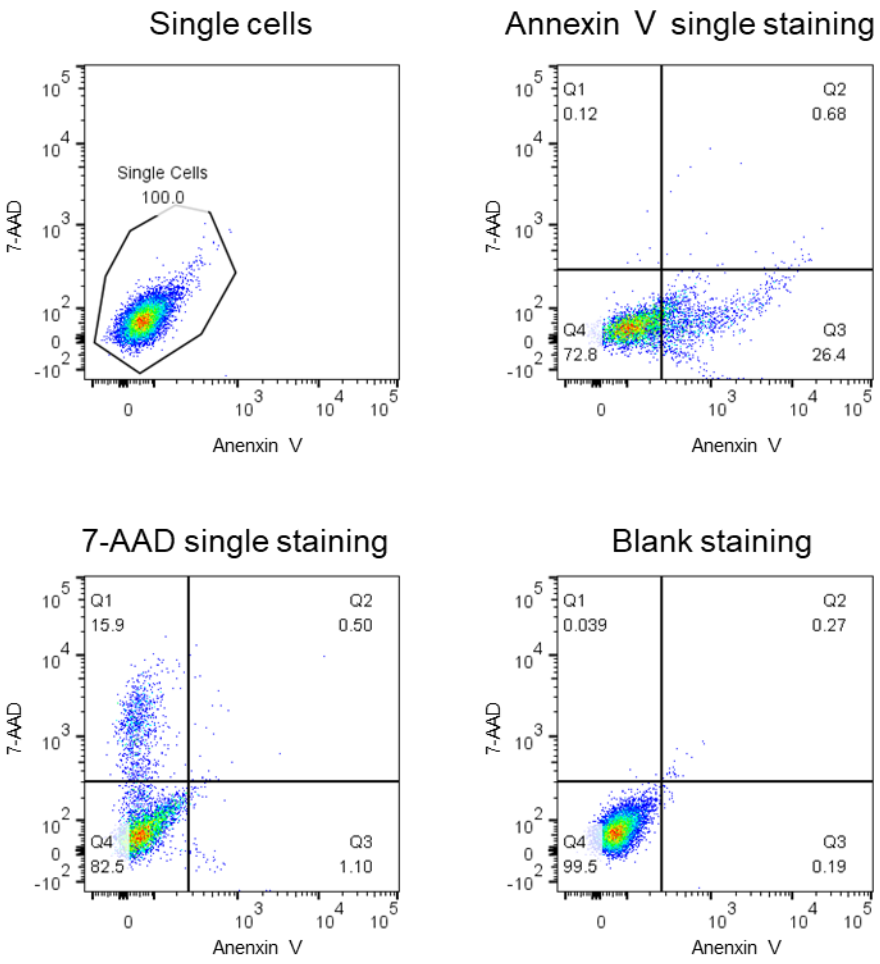

Supplementary Figure 7. Gating strategy for sorting apoptotic cells. For the assessment of apoptosis, cells were trypsinized, washed with 1× phosphate-buffered saline (PBS) and stained using the Annexin V-PE/7-AAD apoptosis detection kit following the manufacturer's instructions. Stained cells and controls (Annexin V-PE only, 7-AAD only and blank) were then processed by fluorescence-activated cell sorting (Cytek Dxp Athena flow cytometer) and analyzed by FlowJo software. Each experiment was repeated three times with similar results, with one representative experiment being shown.

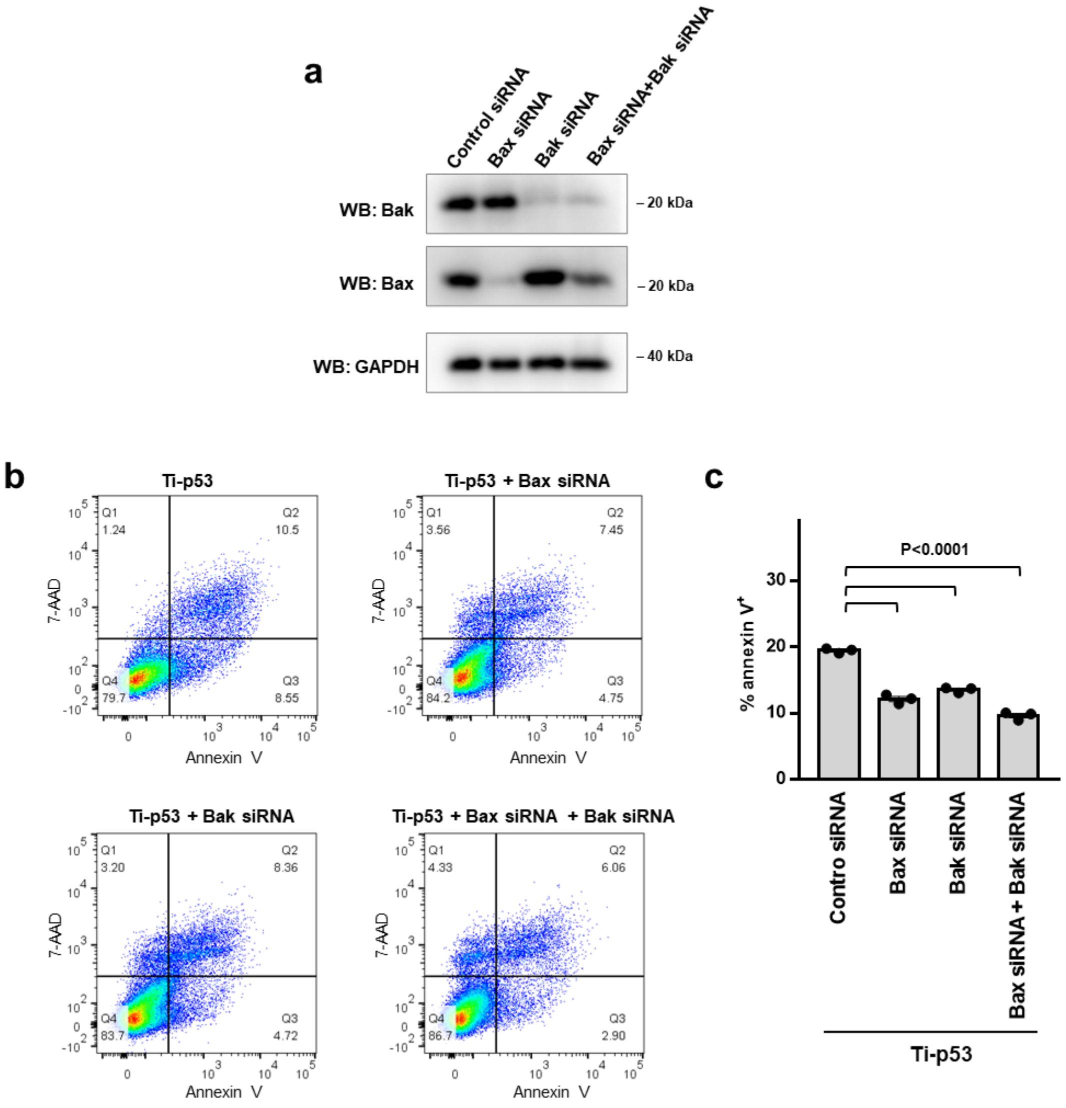

Supplementary Figure 8. Knockdown of Bax and/or Bak attenuates Ti-p53-mediated apoptosis. HCT116 cells with stable Ti-p53 expression were transfected with Bax and/or Bak siRNAs for 48 hours as indicated. **a.** Western blotting was used to detect the transfection efficiency of Bax and/or Bak siRNAs. **b-c.** Flow cytometry was used to determine the effect of Bax and/or Bak siRNAs on the apoptosis ratio. Cells were stained with Annexin V-PE/7-AAD before being subjected to flow cytometry. One representative experiment is shown in (**b**), as three independent replicates were similar. Data are represented as the mean ± SEM of n=3 independent experiments (**c**). P values were determined by one way ANOVA followed by Dunnett's multiple comparisons test. Source data are provided as a Source Data file.

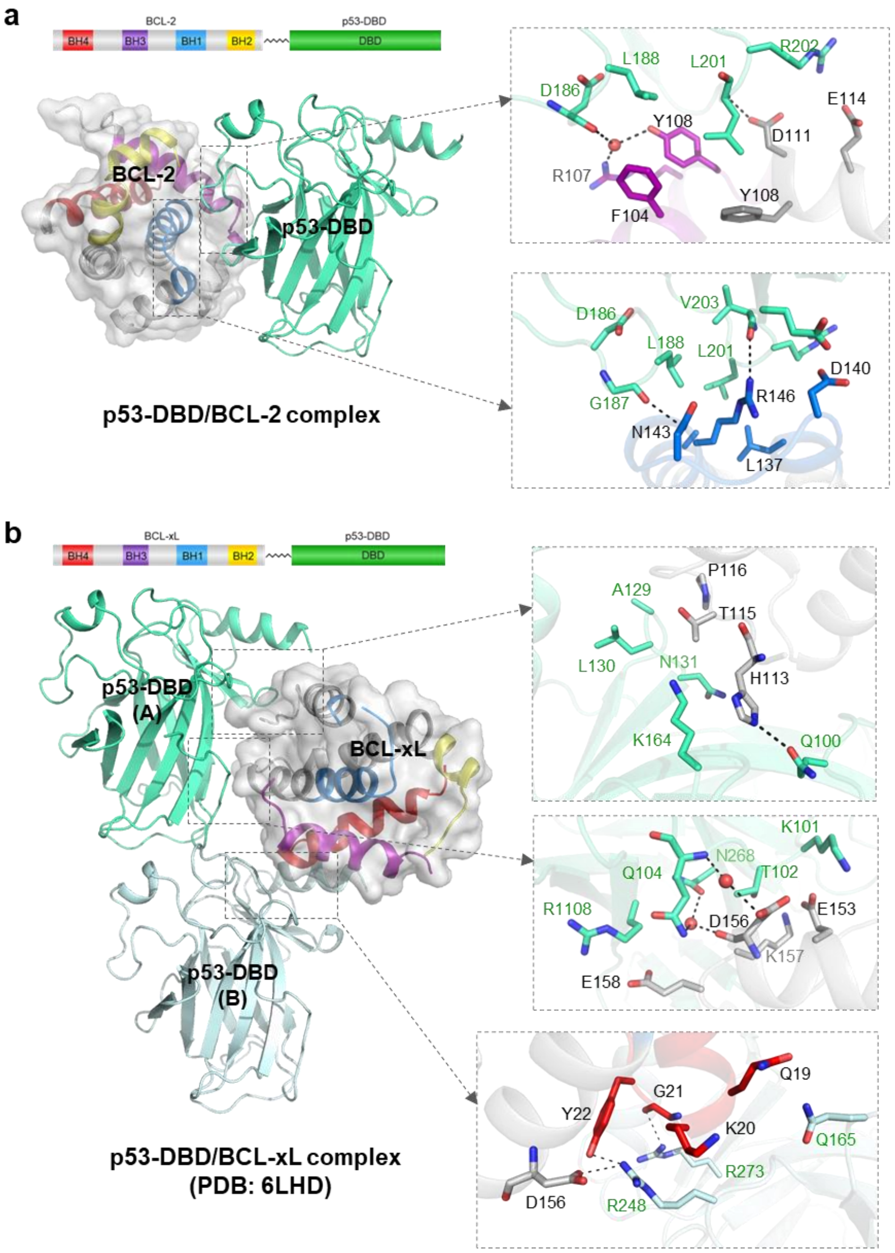

Supplementary Figure 9. Structural comparison of the p53-DBD/BCL-2 complex with the p53-DBD/BCL-xL complex. **a**. Structure of the p53-DBD/BCL-2 complex. p53 is shown as green cartoons. BCL-2 is shown as gray cartoons and surfaces with BH1-BH4 domains colored blue, yellow, purple and red, respectively. **b**. Structure of the p53-DBD/BCL-xL complex (PDB: 6HLD). One p53-DBD molecule (A chain, colored green) is superposed to the p53 molecule in the p53-DBD/BCL-2 complex. The other p53-DBD molecule (B chain) is colored light green. BCL-xL is shown as gray cartoons and surfaces with BH1-BH4 domains colored blue, yellow, purple and red, respectively. The detailed interfaces in the two complexes are highlighted in the boxes.

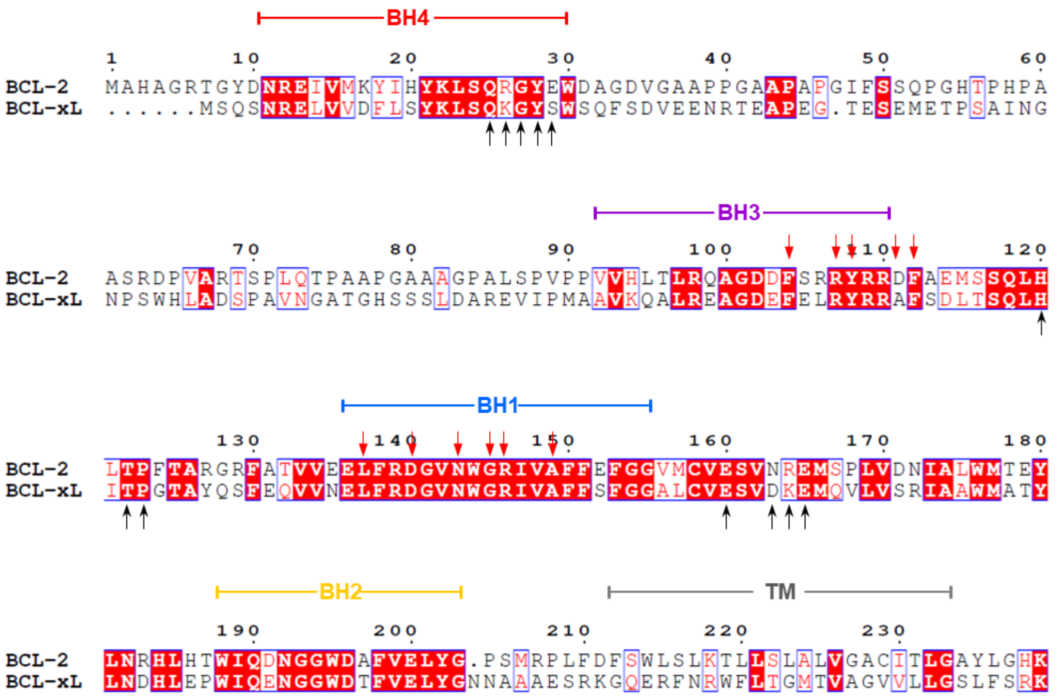

Supplementary Figure 10. Sequence alignment of BCL-2 and BCL-xL. The sequence alignment was created using the web services ESPript 3.0. Identical and similar residues are boxed in red and white, respectively. The BH1-BH4 domains are labelled. BCL-2 residues at the p53-DBD/BCL-2 interface are labelled by red arrows. BCL-xL residues at the p53-DBD/BCL-xL interface are labelled by black arrows.
