# Supplementary material for: Structures of p53/BCL-2 complex suggest a mechanism for p53 to antagonize BCL-2 activity
Source: Nat Commun. 2023 Jul 18;14:4300. doi: 10.1038/s41467-023-40087-2 (PMC10353994; doi:10.1038/s41467-023-40087-2)
Supplement: Supplementary file 3 — Reporting Summary [file 41467_2023_40087_MOESM3_ESM.pdf]

nature portfolio

Corresponding author(s): Yongheng Chen, Ming Guo

Last updated by author(s): Jul 7, 2023

Reporting Summary

Nature Portfolio wishes to improve the reproducibility of the work that we publish. This form provides structure for consistency and transparency in reporting. For further information on Nature Portfolio policies, see our Editorial Policies and the Editorial Policy Checklist.

Statistics

For all statistical analyses, confirm that the following items are present in the figure legend, table legend, main text, or Methods section.

n/a	Confirmed	
☐	☒	The exact sample size (*n*) for each experimental group/condition, given as a discrete number and unit of measurement
☐	☒	A statement on whether measurements were taken from distinct samples or whether the same sample was measured repeatedly
☐	☒	The statistical test(s) used AND whether they are one- or two-sided<br>*Only common tests should be described solely by name; describe more complex techniques in the Methods section.*
☒	☐	A description of all covariates tested
☐	☒	A description of any assumptions or corrections, such as tests of normality and adjustment for multiple comparisons
☐	☒	A full description of the statistical parameters including central tendency (e.g. means) or other basic estimates (e.g. regression coefficient) AND variation (e.g. standard deviation) or associated estimates of uncertainty (e.g. confidence intervals)
☐	☒	For null hypothesis testing, the test statistic (e.g. *F*, *t*, *r*) with confidence intervals, effect sizes, degrees of freedom and *P* value noted<br>*Give P values as exact values whenever suitable.*
☒	☐	For Bayesian analysis, information on the choice of priors and Markov chain Monte Carlo settings
☒	☐	For hierarchical and complex designs, identification of the appropriate level for tests and full reporting of outcomes
☒	☐	Estimates of effect sizes (e.g. Cohen's *d*, Pearson's *r*), indicating how they were calculated

Our web collection on statistics for biologists contains articles on many of the points above.

Software and code

Policy information about availability of computer code

Data collection	G:Box Cheni-XX9 GENESys (Coomassie blue and chemiluminescence imaging), ESPript 3.0 (protein sequence alignment), PerkinElmer Envision Manager (version 1.13.3009.1409, ELISA and FPA data collection), MO.Control (version 2.4.2.16703, MST data collection).
Data analysis	HKL2000 (version 716.1, x-ray data processing), PHENIX (version 1.10.1-2155, structure determination and refinement), WinCoot (version 0.8.9, atomic model building), GraphPad Prism 8.0 (ELISA and FPA data analysis), PyMOL (version 2.3.3, molecular graphics and analyses), MO.Affinity Analysis 3 (MST data analysis),FlowJo CE (version 10, calculating the apoptotic cell rate).

For manuscripts utilizing custom algorithms or software that are central to the research but not yet described in published literature, software must be made available to editors and reviewers. We strongly encourage code deposition in a community repository (e.g. GitHub). See the Nature Portfolio guidelines for submitting code & software for further information.

Data

Policy information about availability of data

All manuscripts must include a data availability statement. This statement should provide the following information, where applicable:

- Accession codes, unique identifiers, or web links for publicly available datasets
- A description of any restrictions on data availability
- For clinical datasets or third party data, please ensure that the statement adheres to our policy

The coordinates and structure factors of the BCL-2/p53-DBD complex generated in this study have been deposited in the Protein Data Bank database under the

accession codes 8HLL, 8HLM and 8HLN. Other data generated in this study are provided in the article and in the Supplementary Information/Source data file. The structure models used in the study are available in the Protein Data Bank under the following accession codes: 2OCJ (structure of apo p53-DBD), 1GJH (apo NMR solution structure of BCL-2), 2XA0 (structure of BCL-2/Bax-BH3 complex), 6QG8 (structure of BCL-2/Puma-BH3 complex), 3KMD (structure of p53/DNA complex) and 6LHD (BCL-xL/p53-DBD structure). A reporting summary is available as a Supplementary Information file. Source data are provided with this paper.

Human research participants

Policy information about studies involving human research participants and Sex and Gender in Research.

Reporting on sex and gender	n/a
Population characteristics	n/a
Recruitment	n/a
Ethics oversight	n/a

Note that full information on the approval of the study protocol must also be provided in the manuscript.

Field-specific reporting

Please select the one below that is the best fit for your research. If you are not sure, read the appropriate sections before making your selection.

☒ Life sciences ☐ Behavioural & social sciences ☐ Ecological, evolutionary & environmental sciences

For a reference copy of the document with all sections, see nature.com/documents/nr-reporting-summary-flat.pdf

Life sciences study design

All studies must disclose on these points even when the disclosure is negative.

Sample size	The sample size was determined based on standards in the field of biochemistry and cell biology, attempting to have a minimum of N = 3 biological replicates with sufficient reproducibility. Sample sizes are stated in the main text figure, figure legend or methods section . For example, MST, FPA and flow cytometry assays consisted of 3 replicates as used in published study (https://www.sciencedirect.com/science/article/pii/S0022286014002750, https://www.sciencedirect.com/science/article/pii/S0003269702000283?via%3Dihub, https://www.nature.com/articles/s41467-021-27210-x).
Data exclusions	No data were exculded.
Replication	For all immunoblotting experiments, three biologically independent replicates were performed with similar results, and a representative experiment is shown. The binding assays (MST, ELISA and FPA) were performed more than three independent experiments, For flow cytometry, three biological replicates were performed and one representative experiment is shown. All replication were successful. Several datasets were collected from different crystals but only the datasets with highest resolution were solved and refined.
Randomization	Samples were allocated randomly.
Blinding	Blinding was not used in this study. Data were derived from instrument-based measurement and software-based analysis.

Reporting for specific materials, systems and methods

We require information from authors about some types of materials, experimental systems and methods used in many studies. Here, indicate whether each material, system or method listed is relevant to your study. If you are not sure if a list item applies to your research, read the appropriate section before selecting a response.

Materials & experimental systems

n/a	Involved in the study
☐	☒ Antibodies
☐	☒ Eukaryotic cell lines
☒	☐ Palaeontology and archaeology
☒	☐ Animals and other organisms
☒	☐ Clinical data
☒	☐ Dual use research of concern

Methods

n/a	Involved in the study
☒	☐ ChIP-seq
☐	☒ Flow cytometry
☒	☐ MRI-based neuroimaging

Antibodies

Antibodies used	Flag-tag antibody (Cell Signaling Technology, 8146T, 1:1000 dilution), HA-tag antibody (Proteintech, 66006, 1:5000 dilution), caspase 3 antibody (Cell Signaling Technology, 9662S, 1:1000 dilution), cleaved caspase 3 antibody (Cell Signaling Technology, 9661S, 1:1000 dilution), cleaved PARP antibody (Cell Signaling Technology, 5625S, 1:1000 dilution), β-actin antibody (Proteintech, 66009-1-Ig, 1:5000 dilution), Bax antibody (Cell Signaling Technology, 2772S, 1:1000 dilution), BCL-2 antibody (Cell Signaling Technology, 15071, 1:1000 dilution), p53 antibody (Abcam, ab26, 1:1000 dilution) for p53-DBD, GST tag antibody (Cell Signaling Technology, 2625S, 1:1000 dilution), and His-tag antibody (Abbkine, ABT2050, 1:5000 dilution)were used. The secondary antibodies HRP-conjugated goat anti-mouse IgG (Abbkine, A21010, ATSDE1601, 1:2000 working dilution) and HRP-conjugated goat anti-rabbit IgG (Absin, abs20040, AS004, 1:2000 working dilution) were used.
Validation	The informations below are according to the manufacturer's website.<br>Anti-p53 antibody (Cell Signaling Technology, 9282S) is a rabbit polyclonal antibody to p53, Mutant p53, Antigen NY-CO-13, LFS1, bbl, and tumor protein p53; it recognizes endogenous levels of total p53 protein, suitable for WB, IP (Immunoprecipitation), chip (Chromatin Immunoprecipitation).<br>Mouse HA-tag antibody (Proteintech, 66006,) is validated by WB analysis in HEK 293 cell.<br>Mouse anti-Flag antibody (Cell Signaling Technology, 8146T) detects exogenously expressed DYKDDDDK proteins in cells, applications in WB, IP, IHC (Immunohistochemistry), chip, IF (Immunofluorescence), Flow Cytometry.<br>Caspase 3 antibody (Cell Signaling Technology, 9662S) is a rabbit polyclonal antibody that detects endogenous levels of full-length caspase-3 (35 kda) and the large fragment of caspase-3 resulting from cleavage (17 kda) from human, mouse, rat and monkey. Has been used in western blot, immunofluorescence and immunoprecipitation.<br>Rabbit cleaved caspase 3 antibody (Cell Signaling Technology, 9661S) detects endogenous levels of the large fragment (17/19 kda) of activated caspase-3 resulting from cleavage adjacent to Asp175.<br>Rabbit Cleaved PARP antibody (Cell Signaling Technology, 5625S) detects endogenous levels of the large fragment (89 kDa) of human PARP1 protein produced by caspase cleavage, suitable for WB, IP, IHC, IF.<br>Mouse β-actin antibody (Proteintech, 66009-1-Ig) is a mouse monoclonal antibody that shows reactivity with human, mouse, rat, hamster, zebrafish, monkey, dog and has been validated for the following applications: FC, IF, IHC, IP, WB, ELISA.<br>Rabbit Bax antibody (Cell Signaling Technology, 2772S) detects endogenous levels of total Bax protein. The antibody does not cross-react with other Bcl-2 family members, suitable for WB, IP.<br>Mouse BCL-2 antibody (Cell Signaling Technology, 15071) recognizes endogenous levels of total Bcl-2 protein, suitable for WB, IP.<br>Mouse anti-p53 antibody (abcam, ab26) has been knockout validated in western blot, and it reacts with mouse or human p53 suitable for ICC-IF, WB.<br>Anti-GST tag mouse monoclonal antibody (Abbkine, 2A8) is validated by Western blot analysis of GST fusion and is suitable for WB.<br>Mouse His-tag antibody (Abbkine, ABT2050) recognizes His; 6 His epitope; Hexa His; HHHHHH epitope; Polyhistidine Tag; application for IF, IP, WB.

Eukaryotic cell lines

Policy information about cell lines and Sex and Gender in Research

Cell line source(s)	HEK293T cells was purchased from ATCC (CRL-3216). p53-/- HCT 116 cells was purchased from Ubigene, YKO-H175.
Authentication	No additional authentication was performed in this study.
Mycoplasma contamination	The cells were tested negative for mycoplasma.
Commonly misidentified lines (See ICLAC register)	None were used.

Flow Cytometry

Plots

Confirm that:

☒ The axis labels state the marker and fluorochrome used (e.g. CD4-FITC).

☒ The axis scales are clearly visible. Include numbers along axes only for bottom left plot of group (a 'group' is an analysis of identical markers).

☒ All plots are contour plots with outliers or pseudocolor plots.

☒ A numerical value for number of cells or percentage (with statistics) is provided.

Methodology

Sample preparation	cells were inoculated onto 6-well plates to ensure cell confluence of 30-50%. After 48 hours, cells were washed with PBS, resuspended in 100 μl of binding buffer. Then, the cells were treated with a Annexin-V PE and 7-AAD staining Kit for 10-15 min in dark at room temperature, and subjected to flow cytometry analysis. A total of 106 cells were analyzed for each sample, and each condition was tested in three independent samples.
Instrument	Cytek Dxp Athena flow cytometer with FlowJo CE software

Software	FlowJo CE software was utilized to calculate the apoptotic cell rate
Cell population abundance	Abundance of relevant cell populations within post-sorted fraction and purity of sorted cell populations were validated from reanalysis by flow cytometry.
Gating strategy	The gating strategy used for determining apoptosis was based on Annexin V-PE and 7-AAD staining. The right edge of the curve was marked as the border of negative and positive.

☒ Tick this box to confirm that a figure exemplifying the gating strategy is provided in the Supplementary Information.
